# Chitosan Supports Boosting NiCo_2_O_4_ for Catalyzed Urea Electrochemical Removal Application

**DOI:** 10.3390/polym15143058

**Published:** 2023-07-16

**Authors:** Fowzia S. Alamro, Mahmoud A. Hefnawy, Sherif S. Nafee, Nada S. Al-Kadhi, Rami Adel Pashameah, Hoda A. Ahmed, Shymaa S. Medany

**Affiliations:** 1 Department of Chemistry, College of Science, Princess Nourah Bint Abdulrahman University, P.O. Box 84428, Riyadh 11671, Saudi Arabia; 2 Chemistry Department, Faculty of Science, Cairo University, Giza 12613, Egypt; 3 Physics Department, Faculty of Science, King Abdulaziz University, Jeddah 21589, Saudi Arabia; 4 Department of Chemistry, Faculty of Applied Science, Umm Al-Qura University, Makkah 24230, Saudi Arabia; 5 Chemistry Department, College of Sciences, Taibah University, Yanbu 30799, Saudi Arabia

**Keywords:** urea removal, spinel oxide: electrochemical oxidation, nickel cobaltite

## Abstract

Currently, wastewater containing high urea levels poses a significant risk to human health. Else, electrocatalytic methodologies have the potential to transform urea present in urea-rich wastewater into hydrogen, thereby contributing towards environmental conservation and facilitating the production of sustainable energy. The characterization of the NiCo_2_O_4_@chitosan catalyst was performed by various analytical techniques, including scanning electron microscopy (SEM) and X-ray photoelectron spectroscopy (XPS). Furthermore, the activity of electrodes toward urea removal was investigated by several electrochemical techniques. As a function of current density, the performance of the modified NiCo_2_O_4_@chitosan surface was employed to remove urea using electrochemical oxidation. Consequently, the current density measurement was 43 mA cm^−2^ in a solution of 1.0 M urea and 1.0 M KOH. Different kinetic characteristics were investigated, including charge transfer coefficient (α), Tafel slope (29 mV dec^−1^), diffusion coefficient (1.87 × 10^−5^ cm^2^ s^−1^), and surface coverage 4.29 × 10^−9^ mol cm^−2^. The electrode showed high stability whereas it lost 10.4% of its initial current after 5 h of urea oxidation.

## 1. Introduction

The oxidation of urea, also known as UOR, presents a promising solution to address energy, environmental, and healthcare challenges. This is attributed to its eco-friendly, cost-effective, and sustainable processing methods [1,2,3]. In conjunction with electrocatalysts, electrochemical methods can potentially enhance molecular conversion on the electrode surface. This process can be facilitated by renewable electricity and can serve the purpose of achieving various objectives such as energy storage and conversion, environmental remediation, and electroanalysis [4,5,6]. Specifically, sewage containing urea has the potential to be transformed into gaseous byproducts using UOR technologies in alkaline environments and producing energy through a well-planned design [7]. Nevertheless, it was observed that urea experienced decomposition primarily into N_2_ and CO_2_ when subjected to an acidic environment using an applied potential exceeding 1.7 V relative to the normal hydrogen electrode (NHE) [3]. When the electrolyte maintains a neutral state, the decomposition of urea primarily yields nitrite and nitrate ions, leading to the generation of CO_2_ [2].

Electrochemical systems have been employed in diverse contexts with distinct arrangements and objectives, leading to heterogeneous urea conversion patterns. Although the electrochemical treatment process is currently in its early stages of development, its exceptional characteristics present encouraging prospects for advancement in energy, environment, and healthcare [8,9,10,11,12]. Thus, the electrochemical oxidation of urea can be employed for both urea removal application (wastewater treatment) [13], and fuel cell (energy conversion application) [14].

The surfaces composed of nickel were subject to a significant modification to improve their electronic properties, physical characteristics, and electrochemical activity. As a result, various bimetallic electrocatalysts based on nickel have been documented in the literature as Ni-Mo [15,16], Ni-Co [17,18], Ni-Rh [19], Ni-Mn [20,21], and Ni-Cu [22,23,24].

AB_2_O_4_ spinel oxides, characterized by a basic structure, have been found to possess remarkable chemical and thermal stability, rendering them highly suitable for diverse catalytic applications. The metallic cations A and B are integral components of this class of compounds. Transition metal oxides with a spinel phase are considered the most appealing anodic materials for electrochemical applications [25]. NiCo_2_O_4_, a type of nickel-based spinel oxide, has gained significant attention in various applications such as fuel cells, oxygen evolution reactions, electrochemical sensors, Li-ion batteries, and supercapacitors [26,27,28,29,30].

Chitosan, a derivative of chitin, is frequently employed in diverse applications. Therefore, it is an option for the creation of new chitosan products. These advancements in fermentation technology have allowed the production of chitosan with unique physiochemical characteristics that differ from those found in waste materials. As a result, this presents a promising opportunity to develop innovative chitosan-based products. An alternative to the traditional sources, such as crab shells, is being considered. Chitosan is a frequently utilized material for immobilization purposes owing to its favorable environmental properties, high absorption capacity, notable layer-forming abilities, superior permeability, increased thermal stability, sturdy mechanical strength, biocompatibility, and ease of accessibility [31].

Chitosan has special structural and functional qualities, such as non-toxicity, hydrophilicity, excellent adhesion, biocompatibility, environmental sustainability, antibacterial and antimicrobial characteristics, and non-carcinogenicity. These characteristics make it a very adaptable and widely used chemical in numerous fields [32,33,34,35,36].

Herein, chitosan is employed to boost the activity of nickel cobalt spinel oxide toward urea electrooxidation. The facile synthesis of nickel cobaltite-based composite was used for electrode fabrication. Comparative studies were performed between NiCo_2_O_4_@Chitosan and unmodified NiCo_2_O_4_. The modified electrode was employed as an efficient electrode for electrochemical urea removal. Thus, different electrochemical techniques were used to judge the electrode performance. Additionally, kinetic parameters were calculated to well-understand the electrochemical oxidation process.

## 2. Experimental

### 2.1. Synthesis of NiCo_2_O_4_

The NiCo_2_O_4_ was synthesized by hydrothermal technique. A mixture comprising CoCl_2_.6H_2_O (6 mmol), NiCl_2_.6H_2_O (3 mmol), urea (8 mmol), NH_4_F (25 mmol), and DI water (40 mL) was subjected to magnetic stirring for 30 min after its mixing in a beaker. The solution was introduced into a 50 mL stainless steel reactor with a polytetrafluoroethylene (PTFE) lining. The mixture was subjected to a consistent temperature of 130 °C for 8 h. Following the natural cooling of the reactor to ambient temperature, the sample containing precursors underwent a 30-min ultrasonic cleaning process with deionized water to eliminate any ionic impurities and loose deposition. The crystalline particles of NiCo_2_O_4_ were ultimately acquired through annealing at a temperature of 400 °C in an air environment for 2 h, with a heating rate of 2 °C per minute.

### 2.2. Synthesis of NiCo_2_O_4_ Supported Chitosan

The nickel cobaltite chitosan composite was synthesized by combining a chitosan solution with NiCo_2_O_4_ nanoparticles. 1.5 g of chitosan was introduced to 60 mL of absolute ethanol in a beaker. Subsequently, the mixture was subjected to gentle stirring while gradually increasing the temperature. A quantity of 1.5 g of nickel cobaltite nanoparticles was introduced into the mixture. The temperature of the solution was reduced to the ambient temperature of the surrounding environment. Introducing NiCo_2_O_4_ into the chitosan solution resulted in crosslinking and subsequent encapsulation of NiCo_2_O_4_ nanoparticles. The polyelectrolytic nature of chitosan in acidic environments is attributed to the protonation of its –NH_2_ functional groups. The consequential equilibrium reaction delineates the ionization state.

Consequently, approximately 3 mL of 10% acetic acid was added to the mixture and agitated until the solution exhibited a thick consistency. After ten minutes, the mixture was subjected to filtration and subsequently washed with distilled water. The final composite was dried in an oven, which was maintained at a temperature of 80 °C for 3 h.

### 2.3. Electrode Fabrication

The working electrode was a glassy carbon electrode with a 0.0707 cm^2^ surface area. A gentle emery paper polish was applied after it had been cleansed with ethanol and double-distilled water. The cast solution was then created by ultrasonically dispersing 10 mg of the catalyst powder (NiCo_2_O_4_ or NiCo_2_O_4_@Chitosan) in 0.75 mL of ethanol and 0.25 mL of 5 wt% Nafion for 1 h. The modified electrodes (NiCo_2_O_4_ or NiCo_2_O_4_@Chitosan) were created as follows: 30 µL of catalyst solution was sprayed onto the electrode’s surface and left to dry for 6 h at 60 °C. The Autolab PGSTAT128N was used to conduct all electrochemical experiments. NOVA (Version 2.1, Metrohm Autolab, Utrecht, The Netherlands), an electrochemistry application, fits the impedance spectrum. The counter and reference electrodes were Pt wire and Ag/AgCl/KCl (sat.); respectively. However, NiCo and NiCo@Chit were used to represent the modified electrodes NiCo_2_O_4_ or NiCo_2_O_4_@Chitosan; respectively, and used as working electrodes for urea electrochemical elimination in alkaline medium applications.

## 3. Result and Discussion

### 3.1. Characterizations of Morphology, Microstructure, and Composition

**Figure 1:**
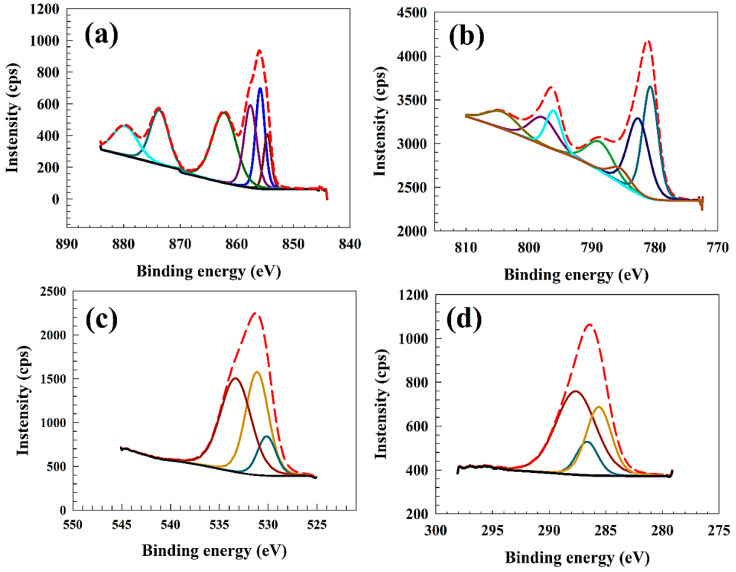
XPS of NiCo nanoparticles (**a**) Ni2p, (**b**) Co2p, (**c**) O1s, (**d**) C1s.

Figure 1a displays the Ni 2p spectrum, which manifests multiple prominent peaks. These peaks are subjected to fitting procedures, which involve the identification of the 2p_3/2_ and 2p_1/2_ peaks and the satellite peaks. The spectral peaks observed at 854.3 and 873.4 eV are attributed to the Ni^2+^ component, whereas the peaks detected at 856.1 and 873.2 eV are associated with the Ni^3+^ component in NiCo, as reported by Hao et al. [37]. The spectral features observed at 863.1 and 879.4 eV are identified as satellite peaks. The spectrum of Co 2p comprises two doublets resulting from spin-orbit coupling and two satellite peaks, as depicted in Figure 1b. The distinctive doublet peaks indicate the presence of the Co^3+^ component observed at 781.2 and 795.2 eV. The characteristic doublet peaks can identify the Co^2+^ component observed at 782.1 and 797.6 eV. The satellite peak observed at 788.6 and 804.2 eV can be attributed to the Co^3+^ and Co^2+^/Co^3+^ components, respectively, as reported by Marco et al. [38]. The spectrum of O1s (as depicted in Figure 1c) can be effectively modeled by three distinct peaks at 530.2, 531.1, and 533.18 eV; respectively. These peaks indicate metal-oxygen bonds, and oxygen defects [39,40,41]. The XPS spectrum of C1s (see Figure 1d), three peaks can be observed at binding energy of 287.1, 286.6, 285.2 eV attributed for C-O, C-N, and C-C; respectively [42,43].

**Figure 2:**
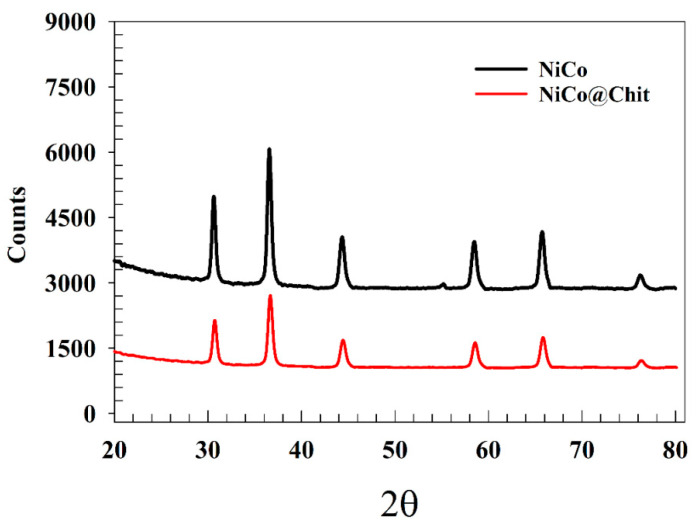
XRD as prepared NiCo, and NiCo@Chit.

The chemical structures of as-prepared NiCo and NiCo@Chit were confirmed using powder X-Ray diffraction technique. Figure 2 shows the XRD chart of as-prepared NiCo_2_O_4_ powder. Thus, several peak observed at 2θ equaled to 31, 37, 44, 58, 65 and 76 that attributed to the reference card of (JCPDS #20-0781) [44]. For the chitosan-based sample, the intensity of the peak decreased because of embedding the nanoparticles in chitosan sheets. The interaction between chitosan and NiCo_2_O_4_ lead to change in lattice structure [45,46,47].

**Figure 3:**
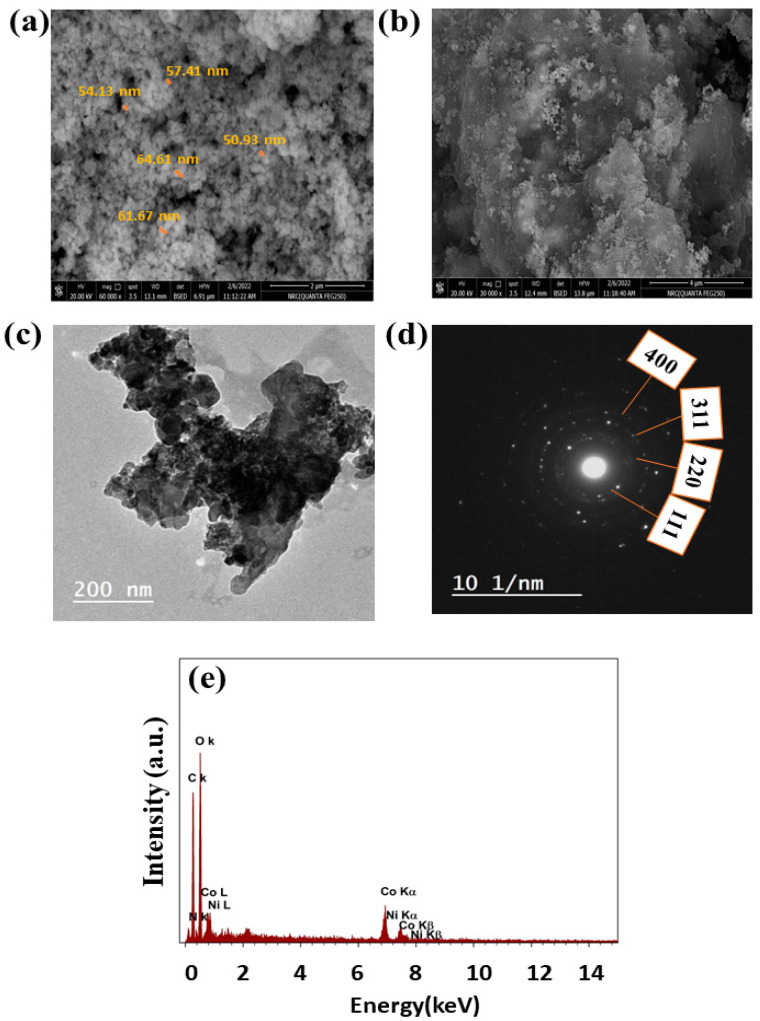
SEM of (**a**) NiCo and (**b**) NiCo@Chit, (**c**) TEM of NiCo@Chit, (**d**) diffraction pattern of NiCo@Chit, (**e**) EDX of NiCo sample.

The morphological characteristics of the NiCo nanostructures in their initial state were examined utilizing scanning electron microscopy (SEM), as illustrated in Figure 3a. The particles ranged in size 35~80 nm. The small particle size of NiCo indicates the higher activity of the prepared materials. Figure 3b shows the NiCo incorporated into the chitosan sheets. The well-distribution of the NiCo on chitosan sheets can explain the electrode’s high electrochemical activity toward urea electrochemical removal. Presence of chitosan can promote urea adsorption.

The conventional method for determining the dimensions of NiCo nanoparticles was the utilization of Transmission Electron Microscopy (TEM). The average particle size of NiCo was approximately ~60 nanometers. Figure 3c shows the TEM of NiCo@Chit. Thus, the nanosphere of NiCo was observed to be attached to the chitosan sheets. The corresponding TEM diffraction patterns are used to confirm the formation of NiCo on the chitosan sheet. As represented in Figure 3d, d-spacing was used to find the Miller indices (hkl) using ImageJ software. However, the observed rings can be attributed to planes of (400), (311), (220), and (111); respectively. The elemental analysis of NiCo@Chit was estimated by EDX. As a result, EDX indicates that Ni, Co, O, C, and N are present. Figure 3e displays the elemental compositions of the NiCo@Chit sample. As a result, the elemental percentages displayed in the inset figure match the target structure of NiCo, which has a Ni/Co ratio of 1 to 2.

### 3.2. Urea Electrooxidation

**Figure 4:**
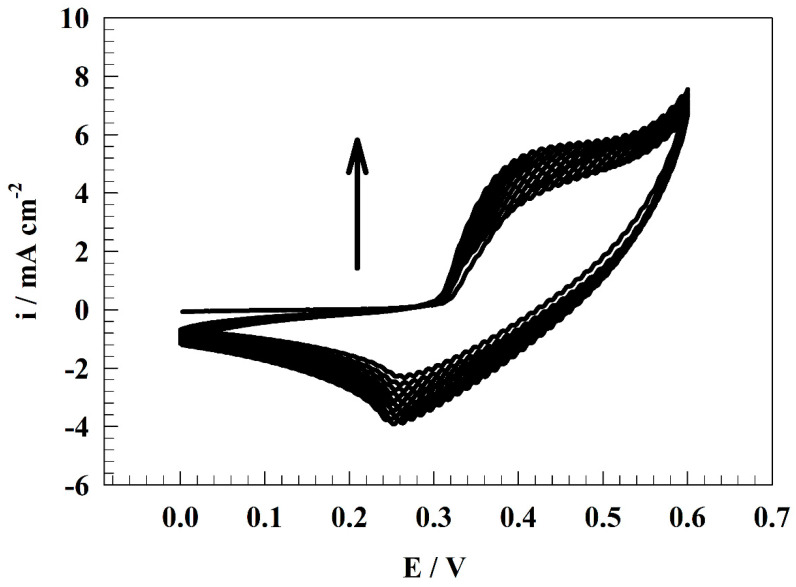
CVs of activation for GC/NiCo@Chit electrode.

The modified GC/NiCo and GC/NiCo@Chit activity was investigated by cyclic voltammetry in a solution of 1.0 M urea and 1.0 M KOH. Activating electrodes composed of nickel is a pivotal stage in the electrochemical oxidation of urea, therefore the electrode performance was enhanced by an activation process; firstly. The outcome of this process is the creation of a Ni-form that exhibits a high degree of electrocatalytic activity, specifically NiOOH. The activation process was executed through cyclic voltammetry (CV) with a scan rate of 100 mV s^−1^ for 150 cycles, utilizing a solution containing 1.0 M KOH(see Figure 4) [48]. The phenomenon of NiOOH formation leads to an increase in current during successive cycles. With an increase in the number of potential sweeps, there is a corresponding increase in the thickness of NiOOH layer. This can be attributed to the presence of OH^−^ ions, which facilitate the rate of conversion between Ni(OH)_2_ and NiOOH according to the following Equation (1) [49,50,51,52]:

(1) $$6\;\mathrm{Ni}(\mathrm{OH}{)}_{2}+6\;{\mathrm{OH}}^{-}\;↔\;6\;\mathrm{NiOOH}+6\;H_{2}O+6\;e^{-}$$

The generated NiOOH species is mainly used for the electrochemical oxidation of urea depending on the following Equation (2):

6 NiOOH+ CO(NH_2_)_2_ + H_2_O ↔ 6 Ni(OH)_2_ + N_2_ + CO_2_ (2)

**Figure 5:**
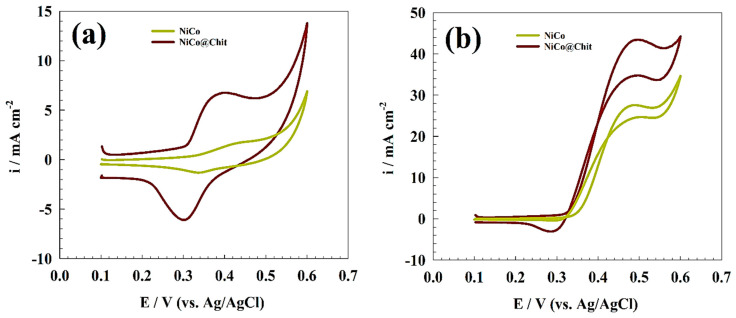
CVs of comparison between NiCo and NiCo@Chit (**a**) in the absence and (**b**) in the presence of 1.0 M urea.

Figure 5a shows CVs of the modified NiCo and NiCo@Chit in 1.0 M KOH. One redox peak can be observed at a potential range of 0.3 to 0.45 V for conversion of Ni(OH)_2_ and NiOOH. Additionally, the urea oxidation can be represented in Figure 5b. Thus, strong oxidation peaks at potential ~0.5 V are attributed to the conversion of urea. However, a sample of NiCo@Chit utilized high activity compared to the unmodified NiCo sample. The presence of chitosan could enhance the activity toward urea electrochemical oxidation in the alkaline medium. The reason for higher activity toward urea electrochemical oxidation may be explained by the ability of chitosan to adsorb urea along with the extended surface area and enhancement of mechanical and chemical stability of chitosan-based samples compared with the unmodified NiCo samples [53,54,55]. Comparative studies between chitosan-based and unmodified NiCo were performed using several approaches. Table 1 summarizes some of the results of the NiCo and NiCo@Chit surfaces.

**Figure 6:**
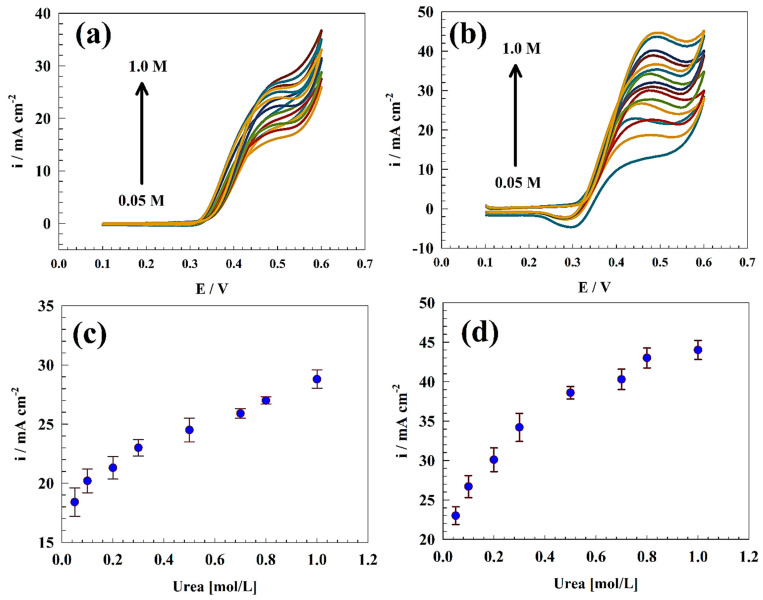
CVs of (**a**) GC/NiCo and (**b**) GC/NiCo@Chit electrodes in 1.0 M KOH and a wide range of urea concentrations. Relation between the urea oxidation current vs. urea concentrations using (**c**) GC/NiCo, and (**d**) GC/NiCo@Chit.

Furthermore, an investigation was conducted on the electrooxidation of urea across a range of concentrations that extend from 0.05 to 1.0 M. Surface saturation was not observed within the concentration range under investigation, as depicted in Figure 6a,b. The specific anodic peak current of the electrooxidation of urea exhibits a positive correlation with the urea concentration (see Figure 6c,d. The results of this study suggest that the electrode under investigation may be suitable for use in applications involving high concentrations of urea, such as in wastewater treatment and direct urea fuel cells (DUFCs). The comparison between the modified NiCo@Chit electrode and others reported in the literature is listed in Table 2.

### 3.3. Urea Oxidation Kinetics

To achieve an in-depth understanding of urea electrochemical oxidation, kinetic parameters were estimated for the oxidation of nitrite over the modified electrodes.

**Figure 7:**
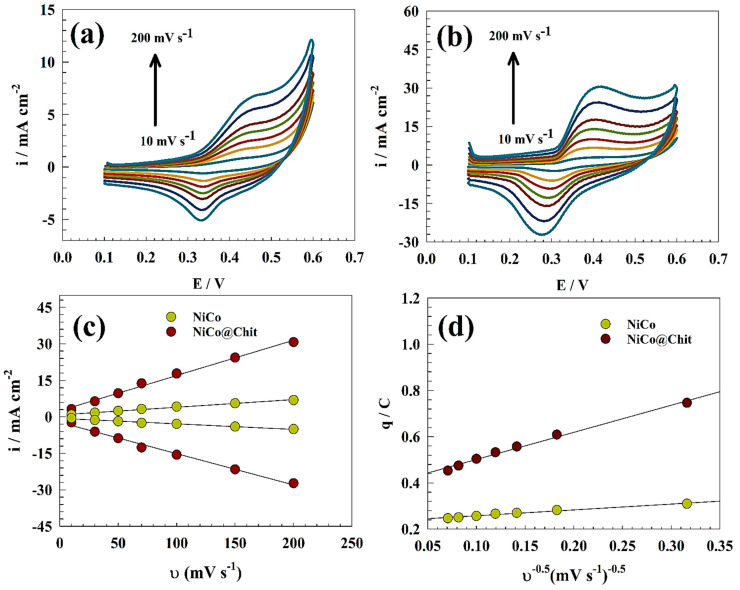
CVs of (**a**) NiCo and (**b**) NiCo@Chit at different scan rates (10 to 200 mV s^−1^) in 1.0 M KOH in the absence of urea. (**c**) Linear relation between redox current versus the scan rate. (**d**) Relation between the charge versus the reciprocal of the square root of the scan rate.

Additionally, various scan rates were used with the modified electrodes NiCo and NiCo@Chit in a solution of 1.0 M KOH as represented in Figure 7a,b. The following Equation (3) was used to estimate the surface coverage:

i = (n^2^F^2^/4RT) A ν Γ* (3)

Where A is surface area, ν scan rate, and Γ* surface coverage, n is the number of electrons, F is the Faraday constant, R is the universal gas constant, and T is the measurement temperature.

As shown in Figure 7c, the relationship between the scan rate and the anodic peak current will reveal the surface coverage. The surface coverage of the modified electrodes, NiCo and NiCo@Chit, was 9.34 × 10^−10^ mol cm^−2^ and 4.29 × 10^−9^ mol cm^−2^; respectively. The larger surface coverage can be observed due to the NiCo@Chit sample’s increased surface activity when urea conversion is compared to unmodified NiCo.

The following relation (Equation (4)) was utilized to confirm that the active sites are evenly dispersed on the surface of the chitosan support electrochemically [60]:

(4) $$q=q_{\infty }+a\;\nu^{-0.5}$$

Where $q_{\infty }$ is the maximum quantity of the charge related to the “outer” surface of active material in Coulombs, q is the charge calculated in Coulombs for various potential scan rates in CV, a is constant (slope of the relation), and ν is the potential scan rate (mV s^−1^) (see Figure 7d).

The intercept value of NiCo@Chit exhibits a significantly higher magnitude than that of pristine NiCo. According to the results, it can be observed that the NiCo@Chit composite possesses active sites that are 1.65 times greater than those of pristine NiCo, thus implying a higher efficiency for urea electrochemical removal. The catalytic reaction is expected to experience significant acceleration on the surface of NiCo@Chit, owing to the abundant active sites of the catalyst that are uniformly distributed and highly effective.

**Figure 8:**
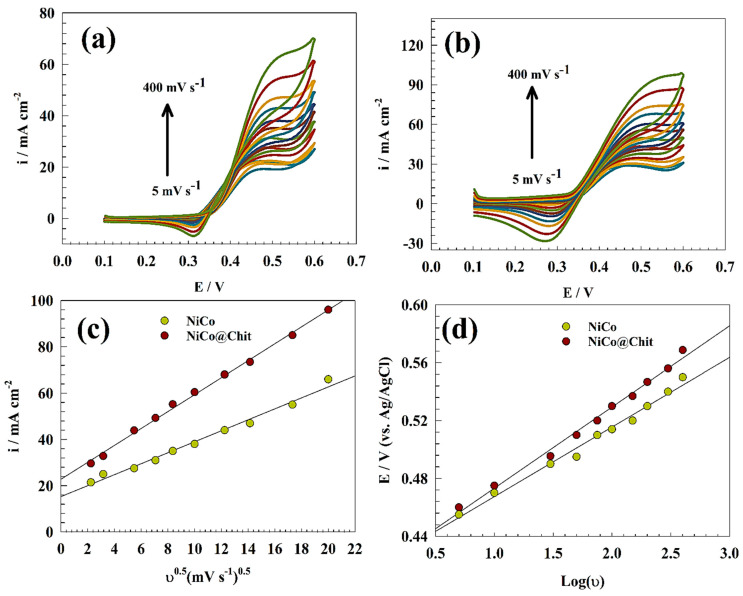
CVs of different modified surfaces (**a**) NiCo and (**b**) NiCo@Chit in a solution of 1.0 M urea and 1.0 M KOH at different scan rates 5 to 400 mV s^−1^. (**c**) Linear relation between anodic current versus the square root of scan rate. (**d**) Linear relation between anodic peak potential versus the logarithmic scan rate.

The CVs of the NiCo and NiCo@Chit modifications were presented in Figure 8a,b; respectively. The measurements were utilized in a solution of 1.0 M urea and 1.0 M KOH, with a scan rate ranging from 5 to 400 mV s^−1^ (vs. Ag/AgCl).

Thus, Randles-Sevcik equation can be employed to calculate the diffusion coefficient (D) for irreversible processes, according to Equation (5) [20,61]:

I_p_ = 2.99 × 10^5^ n A C_o_ [(1 − α) n_o_ D ʋ]^0.5^ (5)

The equation mentioned above denotes the relationship between various parameters, namely the urea oxidation current(i), the number of electrons represented by (n), the surface area of the electrode denoted by (A), the analyte diffusion coefficient represented by (D), the analyte concentration denoted by (C_o_), and the scan rate represented by (ν).

The Randles-Sevick method was employed to estimate the diffusion coefficient. This was achieved by establishing a linear correlation between the current of nitrite oxidation and the square root of the scan rate, as illustrated in Figure 8c. The diffusion coefficients for NiCo and NiCo@Chit electrodes are reported as 5.98 × 10^−6^ and 1.87 × 10^−5^ cm^2^ s^−1^; respectively. The enhanced diffusion coefficient observed for a surface based on chitosan can be attributed to the increased capacity of chitosan to adsorb urea.

Figure 8d illustrates a linear correlation between the peak potential and the logarithm of the scan rate across various modified surfaces. The confirmation of reversibility can be established through the positive shift of the Ep with an increase in the scan rate. The Laviron Equation (6) for irreversible reactions was utilized to observe a change in the location of the peak potential through an increase in the scan rate values [62,63]:

(6) $$E_{\mathrm{pa}}(V)=E{}^{\circ}-\frac{RT}{\propto nF}\;\ln\;\frac{RTk_{s}}{\propto nF}+\frac{RT}{\propto nF}\;\ln\;v$$

The previously mentioned variables, namely E_pa_ denoting peak potential, R representing the universal gas constant, E° signifying formal potential, T indicating temperature, n denoting the number of electrons, v representing scanning rate, and F representing the Faraday constant, are of significance in the academic context.

The transfer coefficient (α) is a kinetic parameter that indicates the propensity of a reaction to proceed in the oxidation/reduction direction. A preference for oxidation direction is observed when the value of (α) is less than 0.5. The transfer coefficients were computed for NiCo and NiCo@Chit using Laviron relation, which involved determining the linear correlation between Log (ν) and E_pa_. The resulting transfer coefficients were 0.46 and 0.53 for NiCo and NiCo@Chit; respectively. The symmetry factor and charge transfer coefficient (α) suggest that urea oxidation on NiCo@Chit has a better reputation than NiCo. However, linear correlation indicates the adsorption of urea onto electrode surfaces.

**Figure 9:**
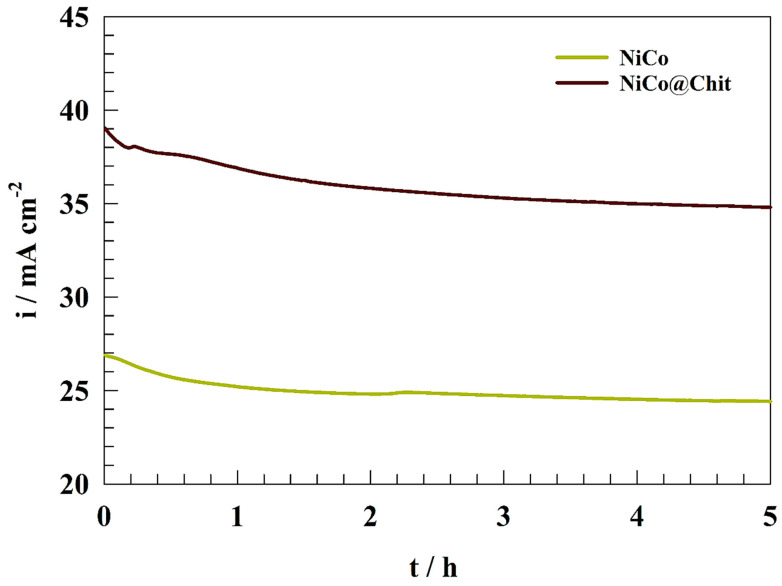
Chronoamperograms of NiCo and NiCo@Chit at constant 0.5 V (vs. Ag/AgCl).

The endurance of the electrode in the face of uninterrupted electrooxidation is the most important in the context of urea elimination. Chronoamperometry was utilized to investigate the enduring stability of the electrode for the electrooxidation of urea. Figure 9 depicts the chronoamperogram of the NiCo and NiCo@Chit-modified electrodes in a solution containing 1.0 M urea and 1.0 M KOH while maintaining a constant oxidation potential of 0.5 V (vs. Ag/AgCl). After 5 h, the electrodes’ oxidation current density exhibited a decrease of 12.3 and 10.4% for NiCo and NiCo@Chit; respectively. The present reduction is attributed to the electrocatalyst surface’s mechanical corrosion, incompletely oxidized urea accumulation, and metal carbonate formation due to the adsorption of the generated carbon monoxide [64]. Nevertheless, minor variations in the oxidation current indicate the enhanced durability of the electrodes to the electrochemical oxidation of urea over an extended period.

**Figure 10:**
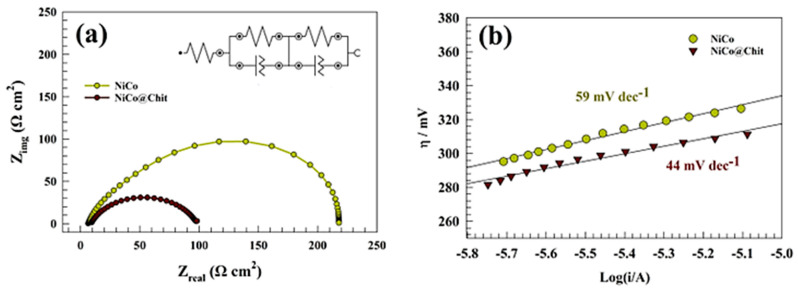
(**a**) Nyquist plots of NiCo and NiCo@Chit surfaces at 0.5 V potential. (**b**) Tafel plots of NiCo and NiCo@Chit.

Electrochemical impedance spectroscopy was employed to ascertain the charge transfer resistance across various electrode surfaces. Figure 10a depicts Nyquist plots of various modified electrodes (GC/NiCo and GC/NiCo@Chit) in a solution containing 1.0 M urea and 1.0 M KOH at 0.5 V (vs. Ag/AgCl). The observation of the double semi-circuit suggests that the process of two-charge transfer warrants consideration. The equivalent fitting circuit corresponding to the statement has been presented in the inset of Figure 10a. The constant phase element (CPE) is employed instead of the capacitive element to account for the non-homogeneity of the electrode surfaces. The resistance values denoted by Rs, R_1_, R_2_, Q_1_, and Q_2_ pertain to the outer and inner layers’ solution resistance, charge transfer resistance, and constant phase element (CPE). Table 3 presents the fitting parameters that were computed. The NiCo@Chit exhibited a charge transfer resistance of 103 Ω cm^2^ while NiCo surfaces displayed a resistance of 230 Ω cm^2^. The enhanced activity of NiCo@Chit in urea oxidation compared to unmodified NiCo can be attributed to the lower charge transfer resistance. However, the EIS data represented in Table 3 confirm the data obtained from the cyclic voltammetry that the modified chitosan composite has higher activity toward urea oxidation due to the high surface area and adsorption ability [55,65].

Figure 10b depicts Tafel analysis employed to investigate the electrochemical kinetics of urea removal by utilizing the Tafel equation. The Tafel slopes computed for the GC/NiCo and GC/NiCo@Chit electrodes are 44 and 29 mV dec^−1^; respectively. The NiCo@Chit sample exhibits a lower Tafel slope, suggesting that the oxidation of urea over the surfaces modified by chitosan is more favorable than the unmodified NiCo surface. The calculated Tafel slopes are comparable with other reported catalysts for urea removal like 22 mV dec^−1^, 21.5 mV dec^−1^, and 26.4 mV dec^−1^ for Ni-MOF, LaNiO_3_, and FeOOH; respectively [11,50,66].

## 4. Conclusions

The present study reports the successful preparation of a Nickel-based spinel oxide (NiCo_2_O_4_) by hydrothermal techniques. The synthesized materials were supported on chitosan sheets to enhance the efficiency of the spinel oxide to electrochemical urea removal.

A comparative analysis was utilized between the performances of pristine NiCo_2_O_4_ versus NiCo_2_O_4_@Chit surfaces. Including functionalized carbon materials in NiCo oxides enhances structural stability, thereby mitigating surface poisoning and ensuring compatibility between the electrocatalyst and glassy carbon surface.

The extended surface area of nickel-containing chitosan enhances its efficacy in facilitating urea removal. NiCo_2_O_4_@Chitosan has been identified as a highly promising material due to its exceptional electrochemical properties. The lower Tafel slopes for chitosan-modified surface indicates the higher thermodynamic favorability.

## Figures and Tables

**Table 1:** Electrochemical parameters for NiCo and NiCo@Chit electrodes.

Electrode	Anodic Current (mA cm^−2^)	Onset Potential (V)	E_pa_ (V)	Tafel Slope mV dec^−1^	Diffusion Coefficient (cm^2^ s^−1^)	Surface Coverage (Γ)/(mol cm^−2^)
NiCo	27	0.35	0.5	44	5.98 × 10^−6^	9.34 × 10^−10^
NiCo@Chit	43	0.32	0.49	29	1.87 × 10^−5^	4.29 × 10^−9^

**Table 2:** Comparison between different surfaces for urea electrochemical oxidation in an alkaline medium.

Electrode	Fuel Concentration (M)	Electrolyte Concentration (M)	Scan Rate (mV s^−1^)	Oxidation Current (mA cm^−2^)	References
NiCo_2_O_4_@Chitosan	1.0	1.0	20	43	This work
Ni_0_._85_Se/rGO	0.5	1.0	50	10	[56]
Ni_0_._9_Cu_0_._1_	0.3	0.5	20	32	[23]
IN738 supper alloy	1.0	1.0	20	12	[57]
NiO-MnOx/Polyaniline	0.3	0.5	20	16	[58]
Ni(OH)_2_ meshes	0.3	1.0	50	20	[59]

**Table 3:** EIS parameters for NiCo and NiCo@Chit electrodes.

Electrode	R_s_	R_1_	Q_1_		R_2_	Q_2_	
	Ω cm^2^	Ω cm^2^	Y_0_	N	Ω cm^2^	Y_0_	m
**NiCo**	3.2	6.76	0.0005621	0.5154	230	0.002180	0.8322
**NiCo@Chit**	2.5	7.56	0.0013547	0.6523	103	0.003715	0.7354

## Data Availability

The data used for research described in this manuscript are available upon request from corresponding authors: shymaasamir80@cu.edu.eg; shymaa@sci.cu.edu.eg (S.S.M); maadel@cu.edu.eg; maahefnawy@gmail.com (M.A.H).
